# Digital health literacy and well-being of students at Sofia University – Bulgaria

**DOI:** 10.1093/eurpub/ckac129.712

**Published:** 2022-10-25

**Authors:** I Patias, K Kaloyanova

**Affiliations:** Department of Computer Informatics, Sofia University, Sofia, Bulgaria

## Abstract

**Background:**

During the Covid-19 pandemic the students in the Faculty of Mathematics and Informatics at Sofia University ‘St. Kliment Ohridski’ applied their abilities for searching health-related information and use it as a factor to improve their well-being.

**Methods:**

The study used the Questionnaire developed by Dadaczynski, K., Okan, O. & Rathmann, K. in 2020 as COVID-19 Health Literacy Survey: University Students (COVID-HL Survey), and the respective Scale Documentation under the Public Health Centre Fulda (PHZF) at the Fulda University of Applied Sciences & Interdisciplinary Centre for Health Literacy Research at Bielefeld University. 216 students, almost 13% (12.78%) of 1.690 students approached, participated.

**Results:**

There is a correlation as our students reported Very easy or easy to the question how easy they deal with the coronavirus information on the Internet - with 71% choosing from all the information they find, 91% using the proper words to find the information, and 71% finding the exact information. Those results were achieved as our students reported feeling over the last two weeks 27.12% very low, 18.08% low, and only 54.80% high (>50) well-being, measuring the dimensions of psychological general well-being by the WHO-5.

**Conclusions:**

Our students reported they can search and retrieve the appropriate information on the coronavirus or related topics, and they are satisfied with the information found. Our students’ abilities to search and retrieve health-related information are applicable even under the pandemic pressure, where they contribute to the improvement of their well-being feelings. [This research is supported by the National Scientific Program “eHealth” in Bulgaria.]

